# The histopathological effects of reabsorbable polyethylene glycol hydrogel (Coseal) on epidural fibrosis in an experimental postlaminectomy model in rats

**DOI:** 10.3906/sag-2009-241

**Published:** 2021-06-28

**Authors:** Emrah KESKİN, Hasan Ali AYDIN, Murat KALAYCI, Emre IŞIK, Utku ÖZGEN, Kenan ŞİMŞEK, Deniz BAKLACI, Mertol GÖKÇE

**Affiliations:** 1 Department of Neurosurgery, Bülent Ecevit University Medical Faculty, Zonguldak Turkey; 2 Department of Pathology, Bülent Ecevit University Medical Faculty, Zonguldak Turkey; 3 Department of Neurosurgery, Atatürk State Hospital, Zonguldak Turkey; 4 Department of Otorhinolaryngology, Bülent Ecevit University Medical Faculty, Zonguldak Turkey; 5 Department of Thoracic Surgery, Bülent Ecevit University Medical Faculty, Zonguldak Turkey

**Keywords:** Polyethylene glycol, surgical sealant, Coseal, epidural fibrosis, failed back surgery syndrome, laminectomy

## Abstract

**Background/aim:**

To investigate the histopathological effects of reabsorbable polyethylene glycol hydrogel (RPGH, Coseal) on epidural fibrosis (EF) following laminectomy in rats.

**Materials and methods:**

A total of 24 rats were equally divided into three groups. In the first group, no treatment was applied after laminectomy (control group, Group 1). In the second group, hemostasis was achieved after laminectomy, and 2 mm absorbable gelatin sponge soaked in saline was placed over the epidural space and the wound was closed (Group 2). In the third group, hemostasis was achieved following laminectomy, and 0.5 mL RPGH (Coseal, Group 3) was squeezed over the dura mater, and the wound was closed. A histopathological examination was undertaken to evaluate arachnoidal invasion and EF.

**Results:**

The results of EF in the Group 2 and Group 3 were significantly lower compared to the Group 1 (p = 0.023 and p = 0.002, respectively). No statistically significant difference was found between the Group 2 and Group 3 in terms of EF (p = 0.957). There was also no statistically significant difference between the mean arachnoidal invasion of the three groups (p > 0.171). However, the rate of arachnoidal invasion was the lowest in the Group 3.

**Conclusion:**

Intraoperative Coseal, a polyethylene glycol polymer, tends to reduce the risk of epidural fibrosis, although this is not statistically significant.

## 1. Introduction

Epidural fibrosis (EF) causes the adhesion of the nerve root to the dura mater as a result of wrapping the epidural space with fibrotic tissue [1]. This fibrotic structure that develops secondary to laminectomy is one of the important causes of low back pain [2,3]. The incidence of pain due to epidural fibrosis (EF) after laminotomy and laminectomy procedures is 1%–40% [4]. Depending on the stages of lumbar disc surgery (removal of disc and bone fragments), clinical conditions, such as low back pain and/or loss of muscle strength (mild or severe) [failed back surgery syndrome (FBSS)] can develop [5–7]. The reported incidence of FBSS ranges from 10 to 40% [8]. The role of EF in FBSS remains controversial due to epidural scar formation in patients who have undergone successful lumbar disc surgery [9]. Despite this contradiction, EF is still considered by many researchers as the most important cause of FBSS. Although many treatment methods (surgical and medical) have been recommended to minimize postoperative scarring, there is not yet a standard treatment algorithm for the prevention and treatment of EF [10–12]. Some studies have reported new methodologies; e.g., anti-inflammatory agents, fat graft, and Adcon-L but no definitive and effective treatment option has been found [13–15].

The resorbable polyethylene glycol hydrogel (RPGH, Coseal) rapidly forms a covalently bonded hydrogel, and thus adheres to both tissue and synthetic vaccine materials. Coseal swells up to four times its volume after administration (in the first 24 h), and the swelling continues as it is reabsorbed. In addition to its use as a sealant in vascular reconstructions, Coseal has been used in cardiovascular surgery in Europe since 2002 for its anti-adhesion effect [16]. In one study, Coseal was found to reduce peritoneal adhesions not only in cardiovascular surgery but after gynecological laparotomy or laparoscopic surgery [17].

In this study, we investigated the histopathological effects of RPGH (Coseal) on EF in a postlaminectomy model in rats due to its anti-adhesion activity, which is not yet used in clinical practice in spinal surgery.

## 2. Materials and methods

This study was conducted at Zonguldak Bülent Ecevit University, Scholl of Medicine, Experimental Research Centre. The ethics review committee evaluated and approved the experimental protocol (2019-20-03/10).

### 2.1. Surgical technique

The same surgical procedure was applied to each rat. Anesthesia was induced using intraperitoneal xylazine (5 mg/kg; Bioveta, Ankara, Turkey) and ketamine hydrochloride (25 mg/kg, Pfizer, İstanbul, Turkey), and spontaneous respiration was maintained. After the backs of the rats were shaved, the surgical area was sterilized with cotton pads soaked in povidone. A sterile cover was placed on the surgical field. A skin incision was made extending between the spinous processes L2 and S1. The L2-S1 distances were revealed by cutting the fascia and muscles bilaterally from the midline. The L3, L4, and L5 lamina were exposed. After the total laminectomy and flavectomy of L3, L4, and L5, hemostasis was achieved using cotton pads, and the dura mater was exposed. No dural defects were observed during these procedures. Electrocautery or bipolar cautery was not used. After laminectomy, muscles, subcutaneous tissues, and skin were sutured, respectively. There were no complications or side effects from the materials used. At four weeks postoperatively, the animals were sacrificed with 200 mg/kg pentobarbital (Bioveta, Ankara, Turkey). All structural components of the vertebral column between T12 and S1, except the skin, were removed. Tissues were transferred to appropriate pathology containers containing 10% buffered formalin solution for a pathological examination.

### 2.2. Groups

A total of 24 *Wistar albino* female rats, weighing 300–350 g, were divided into three equal groups. The animals were kept below a constant temperature (18–21 °C), and adequate nutrition and photoperiod (12-h light/dark cycle) were provided for the duration of the experiment.

The first group received no treatment after laminectomy, and the wound was closed after hemostasis was achieved (control group). In the second group, after hemostasis was achieved after laminectomy, 2 mm absorbable gelatin sponge (Surgifoam 100-C, Ethicon Inc., USA) was soaked in saline (0.5 mL) and placed over the epidural space and the wound was closed. In Group 3, after hemostasis was achieved following laminectomy, 0.5 mL RPGH (Coseal, 2 ml, International Inc., USA) was squeezed over the dura mater, and the wound was closed. No rat died due to the procedures performed during the study, and all animals were able to perform movement functions before sacrifice. Furthermore, no complications were observed after the procedures.

### 2.3. Evaluation and grading of EF

Tissues were fixed in 15% buffered formalin for 1 week and decalcified for 5 days. After obtaining three 2 mm thick sections from the laminectomy area, the sections were embedded in paraffin. Sections of 5 μm were obtained axially and stained with Masson’s trichrome (Ventana, Trichrome Staining Kit, Tucson, Arizona, USA) using an automated slide stainer (Benchmark, Ventana Medical Systems Inc, Tuscon, AZ, USA). The slides were then examined using a microscope (Zeiss Axio Imager 2, Göttingen, Germany) and photographed with a camera (Zeiss AxioCam ERc 5s, Göttingen, Germany). Arachnoidal invasion and EF were blindly evaluated by a pathologist. The grading system of He et al. was used in the evaluation of EF [18]. Mean values ​​were obtained for the statistical evaluation (Table 1).

**Table 1:** Histopathological classification of epidural fibrosis (18).

Grade 0	No fibrosis affecting the dura mater
Grade 1	Only a thin fibrous band between the scar tissue and dura
Grade 2	Adhesion continues to progress but does not cover the entire laminectomy defect (less than 2/3)
Grade 3	Scar tissue adhesion is larger, constituting more than 2/3 of the laminectomy defect and/or extending to the nerve root

### 2.4. Statistical analysis

The Statistical Package for the Social Sciences software, version 22.0 (IBM Corp., Armonk, NY, USA) was used for data analysis. Frequencies as the 25th to 75th percentiles for ordinal and categorical variables were presented for descriptive statistics. The chi-square test was conducted to evaluate the percentile differences between the groups for categorical variables. The expected values were <5 in boxes; thus, Fisher’s exact test was used. Since the data did not show a normal distribution, the Kruskal–Wallis test, also known as Dunnett’s nonparametric multiple comparison, was applied to determine statistical significance based on the average scores of the groups regarding the density of EF. A p value of less than 0.05 was accepted as statistically significant.

## 3. Results

**Figure 1:**
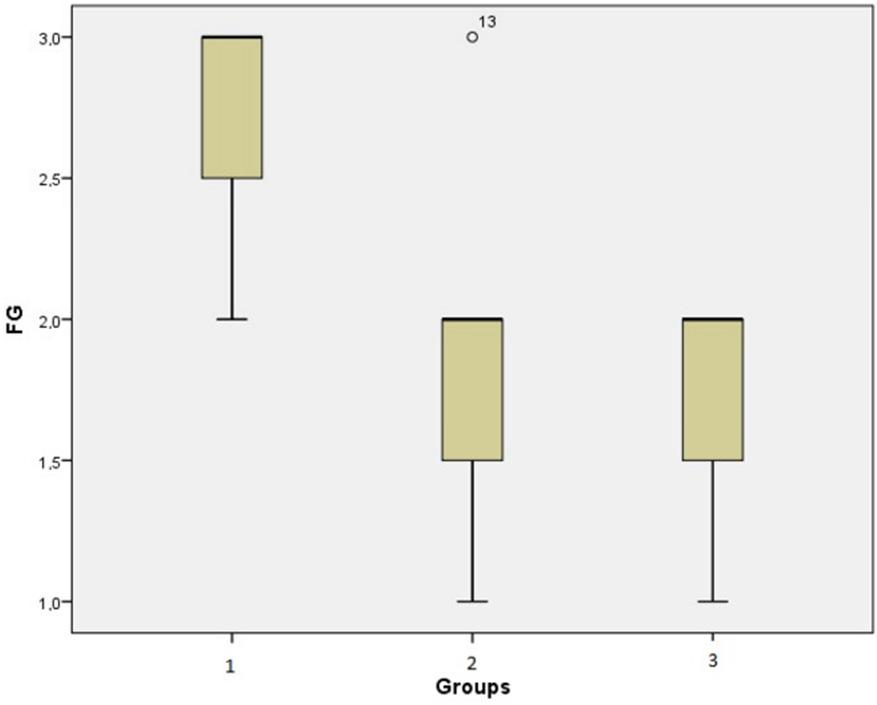
Box plot showing the comparison of the mean fibrosis grades (FG).

**Figure 2:**
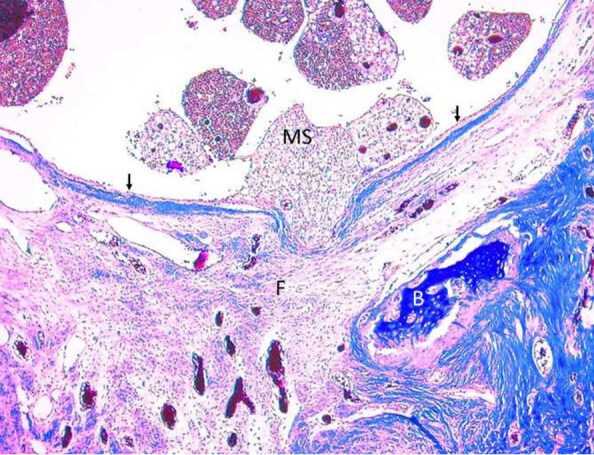
Photomicrograph showing Grade 1 fibrosis in the RPGH group. Thin epidural fibrosis (F) adhered to the dura mater (arrows). (MS) Medulla spinalis; (B) Bone. (Masson’s trichrome, original magnification  100).

**Figure 3:**
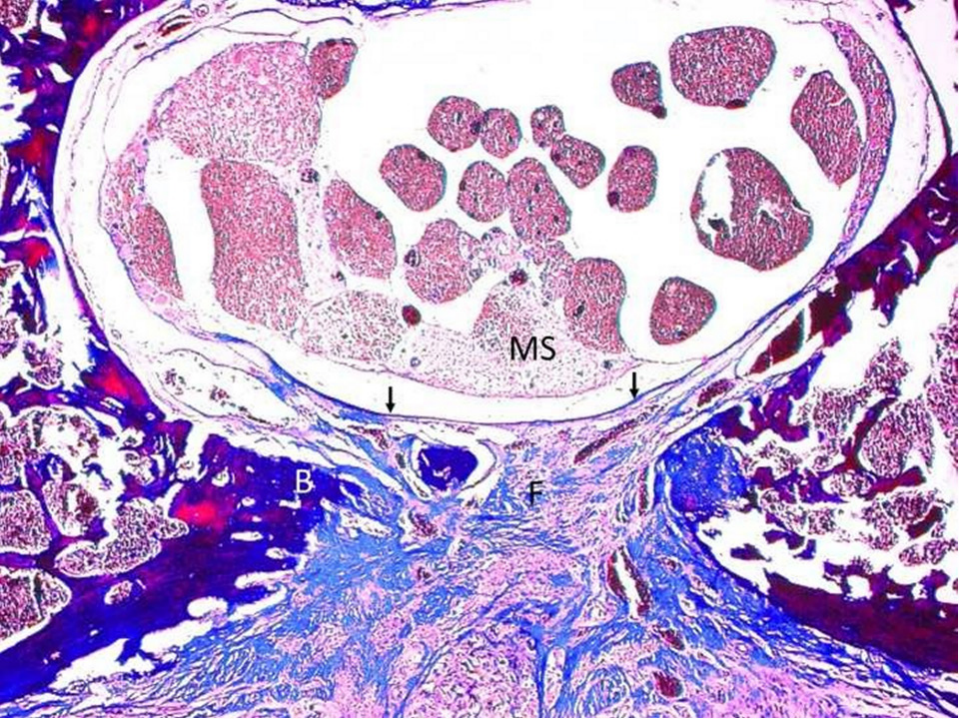
Photomicrograph showing Grade 2 fibrosis in absorbable gelatin sponge group. Epidural fibrosis (F) covered less than 2/3 of the laminectomy defect and adhered to dura mater (arrows). (MS) Medulla spinalis; (B) Bone. (Masson’s trichrome, original magnification  100).

**Figure 4:**
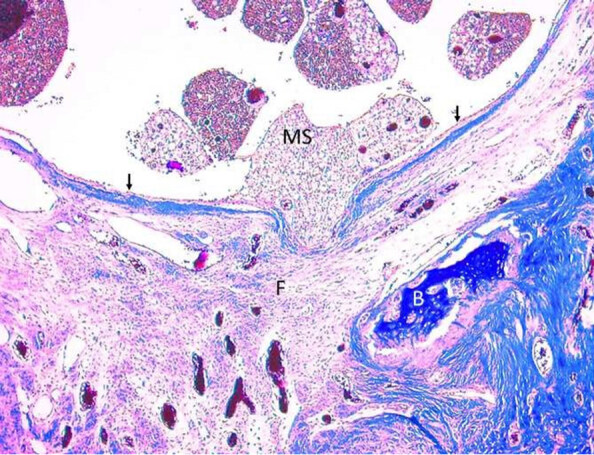
Photomicrograph showing Grade 3 fibrosis in the control group. Epidural fibrosis (F) completely covered the laminectomy defect and adhered to the underlying dura mater (inside, arrow). (MS) Medulla spinalis; (B) Bone. (Masson’s trichrome, original magnification  100).

The rates of EF and arachnoidal invasion in the groups are shown in Tables 2 and 4, respectively. The differences between the groups in terms of fibrosis degrees were found to be statistically significant (p = 0.005; Figure 1). When the effect of this difference was examined based on the homogeneous distribution of variances with the Dunnett post-hoc analysis, it was observed that the results in the Group 2 statistically significantly differed compared to the Group 1 (p = 0.023, Figure 2, 3). Another difference was seen between the Group 1 and Group 3 (p = 0.002). The results of EF in the Group 3 were significantly lower and statistically significant different from the Group 1. On the other hand, there was no significant difference between the average values of the Group 2 and Group 3 (p = 0.957) (Table 3). While 3/4 of the rats in the Group 1 had grade 3 EF, the Group 3 had no rat with grade 3 EF (Figure 4). No statistically significant difference was observed in the mean arachnoidal invasion between the three groups (p > 0.171) (Table 4). However, the rate of arachnoidal invasion was the lowest in the Group 3.

**Table 2:** Fibrosis grades of groups.

Group	Fibrosis Grade	n	%
I	2	2	25.0
	3	6	75.0
II	1	2	25.0
	2	5	62.5
	3	1	12.5
III	1	2	25.0
	2	6	75.0

I: control, II: absorbable gelatin sponge, III: resorbable polyethylene glycol hydrogel (Coseal)

**Table 3:** Differences between the groups according to fibrosis grades (p values*).

Groups	F	I	II	III
I	18.50	-	0.023	0.002
II	10.13	.023	-	.957
III	8.88	.002	.957	-

I: control, II: absorbable gelatin sponge, III: resorbable polyethylene glycol hydrogel (Coseal)*Calculations were performed by the Kruskal–Wallis test. F = mean rank of fibrosis grades. *Significant at the 0.05 level.

**Table 4:** Differences between groups according to arachnoidal involvement.

Groups	(-)	(+)	p value
I	5(%62.5)	3 (%37.5)	
II	6 (%75)	2 (%25)	0.171
III	8 (%100)	0 (%0)	

I: control, II: absorbable gelatin sponge, III: resorbable polyethylene glycol hydrogel (Coseal)* Chi-squared test was used for difference between groups and arachnoidal involvement.(-) no arachnoidal involvement, (+) arachnoidal involvement

## 4. Discussion

The capabilities of an effective anti-adhesion product should not be restricted to preventing adhesion formation following either de novo adhesion or adhesiolysis. It should also demonstrate efficacy and safety in restoration of normal wound healing without causing inflammation while being cost effective to an acceptable degree. Ideally, the must possess properties such as applicability with microscopic and laparoscopic surgical approaches as well as resorbability. Postoperative EF causes persistent back and/or leg pain in patients undergoing lumbar spine surgery [19]. Currently, there is no accepted barrier product or drug in the routine neurosurgery that prevents EF and meets these criteria. This study primarily aimed to evaluate the effects of Coseal on EF and arachnoidal invasion. Our results showed that although there was no statistically significant difference between Group 3 and Group 2, which had no infectious side effects, Group 3 presented with better histopathological data. FBSS is a complex syndrome due to a variety of possible etiological mechanisms and many unknowns, and may lead to the need of reoperation. EF, one of the main causes of FBSS, is still a major debate among neurosurgeons since this complex process involves many factors. Adhesion formation in EF can be explained by the migration of fibroblasts from muscle tissue to the epidural space after laminectomy [16]. It has been observed in the literature that local and/or systemic agents that create a barrier effect in the epidural space and reduce bleeding are recommended to prevent fibrosis [17,20,21,22]. However, despite the significant number of studies in the literature, there is still no clear treatment methodology for EF, which remains an important problem and cause significant discomfort in patients. Other major disadvantages of using similar barriers include foreign body reaction, high costs, infection, and spinal canal compression.

Mitomycin-C is an antibiotic that has an effect on fibroblast proliferation and is generally preferred in ophthalmologic surgery and safely used topically [23]. Doğulu et al. published a paper about the effects of mitomycin-C on postlaminectomy EF using 12 albino rats [24]. They observed a lower degree of fibrosis in the mitomycin-C group. Although the effect of mitomycin-C on fibrosis in rats is statistically significant, studies on humans are needed.

Adcon-L is a gel that can prevent fibroblast migration to the surgical site by creating a physical barrier around the laminectomy area [25]. It is a semisynthetic product that forms a degradable mechanical barrier around the dura and obstruct formation of peridural fibrosis and adhesions after spinal surgery. Doğulu et al., who formed three different study groups, Adcon-L, mitomycin-C, and aprotinin, reported less peridural scar formation in the former two compared to aprotinin [24]. On the other hand, Ganzer applied Adcon-L to 46 patients intraoperatively and closed the wounds without additional application in 46 patients as the control group. After evaluating these 92 patients, the authors did not observe a significant difference in scarring on postoperative magnetic resonance imaging [22].

In some studies published in the literature, hemostatic agents have been used to prevent bleeding that may cause EF in the surgical field. These studies have revealed the anti-inflammatory and anti-fibrotic activities of these agents based on their effects on reducing bleeding in the epidural space [26,27]. LaRocca and Macnab showed that granulation tissue first formed in the epidural area due to hematoma leaking from the traumatized muscles due to surgery, and then this organized structure transformed into EF [28]. Platelet-rich fibrin (PRF), acting as a barrier due to its membranous structure, not only controls fibroblast migration to the wound area but also reduces adhesion by controlling collagen synthesis. Demirel compared the effects of Adcon-L and PRF on EF using 28 *Sprague-dawley* rats and showed that the PRF group developed lower EF than the Adcon gel group [29].

Two commercially available polyethylene glycol (PEG) polymers include vascular sealant (Coseal, Baxter, Fremont, CA) and dural sealant (Duraseal, Covidien, Waltham, MA) [30]. Thanks to the Coseal double syringe spray applicator system, the sealant can be applied from a distance of approximately 3 cm to the surgical dry area after diluting by mixing liquid and powder components for at least 60 s. If there is no gelling after 30 s, the surgical area should be washed with saline and the components should be removed from the area. The tip of the applicator system must be cleaned after each application to avoid clogging [30]. In our study, Group 3 rats were received as a thin layer on the dura by adhering to these procedures. Coseal differs from human or animal originated sealants with its synthetic feature. It consists of multifunctional PEG chains crosslinked to strongly adhere to the area of application [31,32] It contains hydrogen chloride and sodium phosphate–sodium carbonate in addition to the two PEG polymers. It is a surgical sealant that can be applied easily, dries quickly and adheres by taking the form of the tissue it is applied to [33]. This synthetic sealant can be applied not only directly to tissue but also with grafts. Coseal can swell up to 400% on the applied surface, and may create a safety problem by creating pressure. It has been reported that pressure-induced nerve damage may occur due to overdose (maximum 16 mL) in confined areas; skin sensitivity in animals has also been observed [30]. None of the rats in our study presented with nerve damage or allergic reactions.

Experimental and clinical studies performed with Coseal to prevent adhesions that occur in cardiovascular and abdominal surgeries have reported favorable outcomes [33–40]. In the experimental cardiac adhesion model for pericardial adhesions, Coseal reduced adhesion formation compared to surgical control and Tissucol and has been reported to be safe [34]. In an experimental rabbit laparotomy model conducted by Quinino et al., the small intestine side, where Coseal coated polypropylene mesh was administrated, showed lower rate of adhesion formation when compared with the side that received uncoated mesh [33]. In this study, it was determined that Coseal was completely absorbed within 1 month as expected and there was no residue in microscopic examination. Park et al. have reported an absence of foreign body reaction and no immune response to Coseal in an experimental laparoscopic nephrectomy model [35]. Slezak compared the effects of BioGlue (originating from bovine serum albumin) and Coseal in a rabbit aorta model and reported that Coseal produced significantly less inflammatory reaction and resulted in significantly less residual material two weeks after administration compared to BioGlue [36]. With these results, it has been found that Coseal is safer than other sealants. In a multicenter, randomized, single-blind study of 71 patients conducted by Mettler et al., the anti-adhesion effect of Coseal in myomectomy surgery was clinically evaluated for the first time [37]. This study showed that Coseal is a barrier that provides promising outcomes in reducing adhesions after myomectomy operations performed both with open and laparoscopic surgery.

In addition to its use as a sealant in vascular reconstructions, Coseal has been used in cardiovascular surgery in Europe since 2002 for its anti-adhesion effect Natour also evaluated the effects of Coseal on anastomic closure of the aorta in a total of 102 patients [38]. In the sealant group (polyethylene glycol sealant, Coseal), the postoperative drainage volumes significantly decreased, and the duration of intensive care and hospital stay were reduced accordingly. In a controlled randomized multicenter trial performed with 148 patients in 2002, Coseal was compared with Gelfoam/thrombin in terms of their efficacy in closing the suture hole along vascular anastomosis line. Despite the equivalent results of both sealants, Coseal achieved the desired effect in a significantly faster time frame [39]. In pediatric cardiac surgery, reopening of the sternum due to staged surgery is an important problem that can cause catastrophic bleeding. Kim et al. have used Coseal in 16 pediatric cases undergoing cardiac surgery and achieved favorable results both as a sealant in the first operation and in prevention of adhesions in the next operation [43]. Despite these successful results in vascular surgery, it was not as effective as fibrin glue in adhering to the kidney surface and preventing urine leakage in the experimental model of laparoscopic partial nephrectomy [41].

DuraSeal (Covidien, USA) is used against cerebrospinal fluid fistulas in addition to standard dural repair methods and as an adhesion barrier to prevent postoperative EF [42–44]. However, side effects such as wound infection, kidney damage, foreign body reaction, neurological problems, and allergic reactions have been reported [30]. Preul et al. applied DuraSeal Xact, a synthetic product containing PEG and trilysine (essential L-lysine amino acid), in a canine laminectomy model [45]. In this experiment, randomly selected dogs were assigned to two hydrogel groups and one control group, each consisting of five animals. In the hydrogel groups, the DSX gel was applied to the epidural space with two separate applicators (Dual Liquid or MicroMyst spray). The author concluded that epidural adhesions were observed less in the hydrogel groups in the examination performed at four months postoperatively, but this result was not statistically significant compared to the control group [45].

In summary, our study is possibly the first to examine the effects of Coseal on EF; however, the absence of a sham group is a limitation. Although our findings are not promising in terms of preventing peridural adhesions and arachnoidal invasions, more comprehensive clinical and/or experimental studies are needed. As the advantage of Coseal, it can be mentioned that it does not prolong the duration of the surgery due to its ease of application via traditional and microsurgical spraying and to immediately drying in the area of application.

## 5. Conclusion

Although there are some studies about the effects of Coseal on adhesion and hemostasis in cardiac and abdominal surgery, there are no randomized controlled studies investigating the effects of Coseal on EF. In this study evaluating the histopathological effects of Coseal on EF using a postlaminectomy model in 24 rats, no statistically significant difference was observed in the Coseal group compared to the spongostan group. However, the EF class has never been observed, and therefore more comprehensive clinical and experimental studies are needed to support the use of Coseal for EF in the future. 

## Ethical approval

All procedures performed in studies involving animals were in accordance with the ethical standards of the institution or practice at which the studies were conducted. This article does not contain any studies with human participants performed by any of the authors.
